# Ergonomic Design and Assessment of an Improved Handle for a Laparoscopic Dissector Based on 3D Anthropometry

**DOI:** 10.3390/ijerph20032361

**Published:** 2023-01-29

**Authors:** Yujia Du, Rui Jiang, Haining Wang

**Affiliations:** School of Design, Hunan University, Changsha 410082, China

**Keywords:** laparoscopic dissector, handle design, 3D anthropometry, dynamic hand position, ergonomic assessment

## Abstract

Laparoscopic surgery (LS) has been shown to provide great benefits to patients compared with open surgery. However, surgeons experience discomfort, low-efficiency, and even musculoskeletal disorders (MSDs) because of the poor ergonomic design of laparoscopic instruments. A methodology for the ergonomic design of laparoscopic dissector handles considering three-dimensional (3D) hand anthropometry and dynamic hand positions was addressed in this research. Two types of hand positions for grasping and stretching were scanned from 21 volunteers using a high-resolution 3D scanner. The 3D anthropometric data were extracted from these 3D hand pose models and used to design an improved handle (IH) that provides additional support for the thumb, a better fit to the purlicue, and a more flexible grasp for the index finger. Thirty subjects were invited to evaluate the IH in terms of muscular effort, goniometric study of motion, and efficiency and effectiveness during four trials of a laparoscopic training task. Questionnaires provided subjective parameters for ergonomic assessment. Positive results included less muscle load in the trapezius as well as significant but small angular differences in the upper limb. No significant reduction in the trial time and no increased percentage of the achievement were observed between the IH and the commercial handle (CH). Improved intuitiveness, comfort, precision, stability, and overall satisfaction were reported. IH provides significant ergonomic advantages in laparoscopic training tasks, demonstrating that the proposed methodology based on 3D anthropometry is a powerful tool for the handle design of laparoscopic dissectors and other surgical instruments.

## 1. Introduction

Laparoscopic surgery (LS) has been widely used for many surgical interventions instead of an open approach due to the benefits for patients, such as a faster recovery and less complicated wound healing [1,2,3], as well as the cost savings for health care systems [4,5,6,7]. However, awkward postures of the upper limb joints caused by looking into a monitor without directly touching the internal organs [8] are imposed on laparoscopic surgeons, which brings physical injuries, mental fatigue, and stress [9]. Discomfort or aches in the shoulders, wrists, palms, and fingers, in addition to nerve function impairment, has been documented by the authors of [10,11], which may lead to musculoskeletal disorders (MSDs). Many of the injuries are associated with maintaining certain prolonged postures during surgical interventions [12,13,14,15] and the deficiencies in the ergonomic design of instruments used for LS [2,16,17,18,19].

Many researchers have claimed the importance of ergonomic principles in laparoscopic instrument design [9,11,20,21]. The poorly crafted design of laparoscopic instruments, especially the handles, can have a deleterious effect on surgeons’ efficiency and well-being [6,9]. The optimal design of the handles of laparoscopic tools has been the object of studies for several decades. Veelen et al. [16] provided a new design of a scissor handle for dissection only and claimed advantages in terms of specialized functional improvements, without being limited by attempts to find solutions to the conflicting requirements of other functions. Shimomura et al. [22] focused on the effectiveness of the general grip of the ring handle by increasing the contact area and bringing the finger insertion angle more perpendicular to the finger midline. Sancibrian et al. [7] developed a new design of a pistol handle by kinematic analysis and ergonomic simulation with a hand model. The study conducted by the authors of [23] involved documentation analysis including information relating to dimensions, shapes, etc., and the identification of all of the improper ergonomic conditions for a thumb-activated handle. The authors of [24,25,26] presented a new laparoscopic grasper handle design based on the size of each surgeon’s hand and analyzed the anthropometric relationship between the hand and the handle size. Critical hand anthropometric parameters were also used in designing a modified ring-type handle [8]. All in all, the design methods of surgical instrument handles in previous studies have involved mostly forward engineering based on documentation analysis.

Although hand dimensions have been included in some of the design processes mentioned above, motion analysis is usually conducted only with a qualitative method or under evaluation. Moreover, tool manufacturers are limited by the available anthropometric hand data, often only using measurements from a single hand position on mold geometries [27,28], which can result in poor comfort and usability in a clinical setting. For laparoscopic instruments, the motion of stretching and grasping, related to opening and closing the end-effectors of the forceps, requires a large force and can cause muscle fatigue after long-term use. In addition, the use of a spring brings extreme postures of the fingers, wrists, and forearms, which could cause neuralgia and neuritis [1,29,30,31]. Therefore, dynamic hand positions of specified motions when working with the handles should be precisely considered during the design procedure in order to promote the usability of laparoscopic dissectors and reduce the MSD of the surgeons.

Several studies have presented the application of three-dimensional (3D) anthropometric data in the design of hand wearables. The authors of [27,32] proposed that capturing functional hand dimensions and scanning dynamic hand positions was a necessary protocol for the design of gloves and tools. Griffin et al. [33] developed a process and special considerations for 3D hand scanning that could help to conduct more robust 3D anthropometric studies for the hand, as related to product design. Yu et al. [34] proposed a hand surface-scanning method to acquire measurements with higher precision and better repeatability compared with manual methods. Chu et al. [35] established short thumb orthoses using 3D anthropometric data collected via a scanning device to achieve a good fit and the absence of pressure areas in clinical practice. Compared with the applications of two-dimensional (2D) anatomical dimensions [6,8,24,36], design methodologies based on 3D hand anthropometry have additional advantages in terms of an ergonomic and customized design, and this is still a largely under-explored domain for the handle design of tools used in LS.

The necessity of ergonomic analysis for the feasibility of surgical instruments has been addressed in previous studies [6,9,37]. Specific methods providing a more reliable and credible evaluation are required for LS based on ergonomic criteria [7,9]. Surface electromyography (sEMG) has been widely used to observe the muscle activity of the upper limb when working with laparoscopic tools [5,6,22,23,38,39,40]. A goniometric study was conducted to analyze the neutral and extreme motions of the wrist, hand, forearm, and upper arms [5,7,16,23,26,38]. Surgical task performance was tested via task execution times, achieved percentage, and the number of failures [22,23,26,31,38,40,41]. Subjective questionnaires with respect to satisfaction with the tools used in surgical training tasks have been used to investigate users’ preferences [5,7,8,23,24,26,38,40]. In addition, establishing the ideal experimental conditions in which surgeons can perform their tasks and obtain the necessary information regarding the instruments is essential for the ergonomic research of laparoscopic tools.

Several studies have been conducted to show that pistol-type handles cause less muscle exertion and less difficulty in completing tasks compared to ring-type handles [5,6,7,23,37,40,41]. A larger contact area has been proven to reduce the discomfort and pain in the palm and fingers [8,22,37,42,43,44]. Moreover, the ergonomic criterion of avoiding extreme hand postures has been evaluated in previous studies [8,16,23,37,45,46].

The main objective of this research was to present a new handle of a laparoscopic dissector based on 3D hand anthropometric data of dynamic hand positions through reverse engineering. The function of laparoscopic dissectors is to spread and dissect tissue, which requires both power grasping and stretching. Positions for the maximal range of hand movements with respect to opening and closing the end-effector were 3D scanned, and the point cloud data obtained were processed and converted to non-uniform rational basis spline (NURBS), which could be applied directly in computer-aided design (CAD) software for the handle design. The main novelty of the handle lies in the methodology by which it was designed, considering several hand positions related to specific operations. Ergonomic assessment of the new handle was conducted using objective and subjective methods including muscular effort, goniometric study of motion, and efficiency and effectiveness, as well as questionnaires.

## 2. Materials and Methods

### 2.1. Handle Design

#### 2.1.1. Apparatus

**Figure 1:**
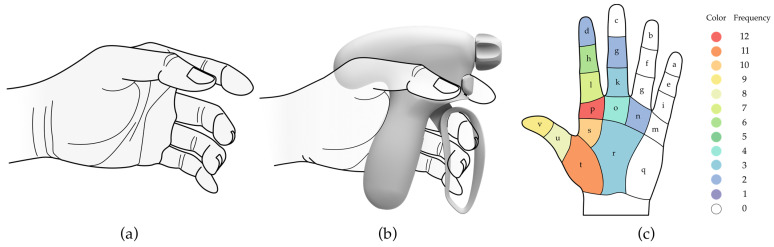
(**a**) Diagram of the natural grip posture of the hand; (**b**) Diagram of the grip method of the OH; (**c**) The frequency of each region considered uncomfortable during the use of the OH.

As shown above, ergonomic-specific requirements were derived for the hand tool design. First, pistol-type handle was adopted for the design of the original version since this type could more easily exert force during the dissection tasks requiring a power grip for grasping and separating body tissue. Secondly, in order to avoid generating small high-pressure zones between the contact area of hand and handle surface, hand surfaces were used to provide a better fit interface for the handle. Considering not appearing awkward hand postures, the natural grip posture of the hand was scanned to refer to the morphology of the handle. After considering several potential designs, the concept presented in Figure 1 was designed by a multidisciplinary group of processionals, which was the original handle (OH) prototype used in this research. The OH was designed for the non-dominant hand, and its pistol-shaped surface was fitted to the natural grip posture of the fingers and palms (Figure 1a), which can provide a larger contact area and enable a comfortable posture without requiring a grasping and stretching motion.

#### 2.1.2. 3D Hand Anthropometric Data Collection

Although the OH was designed based on the natural grip position of the hand, negative feedback on comfort and usability was collected from 5 surgeons and 7 designers in terms of the operation of grasping and stretching. The frequency of each region (*a* to *v*) that was considered to be uncomfortable is shown as Figure 1c. The improved handle (IH) prototype was produced after a design process of reverse engineering. To reduce the muscle load of the fingers and the palm, three design requirements were fulfilled during the design process, (1) additional support for the thumb, (2) a better fit for the purlicue, and (3) more flexibility for the index finger.

**Figure 2:**
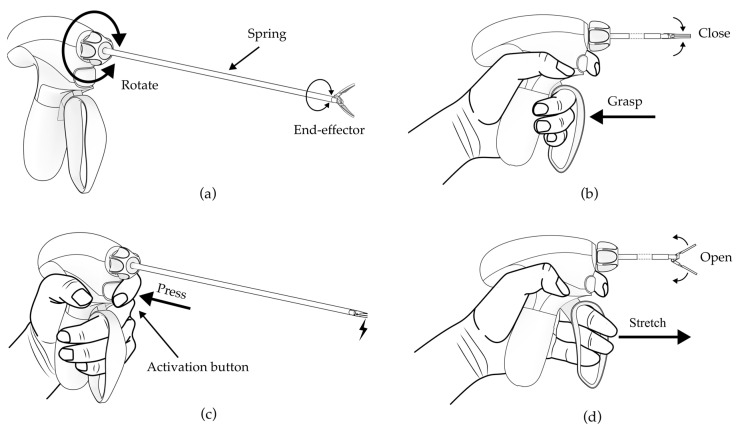
(**a**) Activation of the degrees of freedom of the dissector; (**b**) Fingers grasp to close the end-effector; (**c**) Index finger presses the activation button to emit energy when the hand is grasping; (**d**) Fingers stretch to open the end-effector.

**Figure 3:**
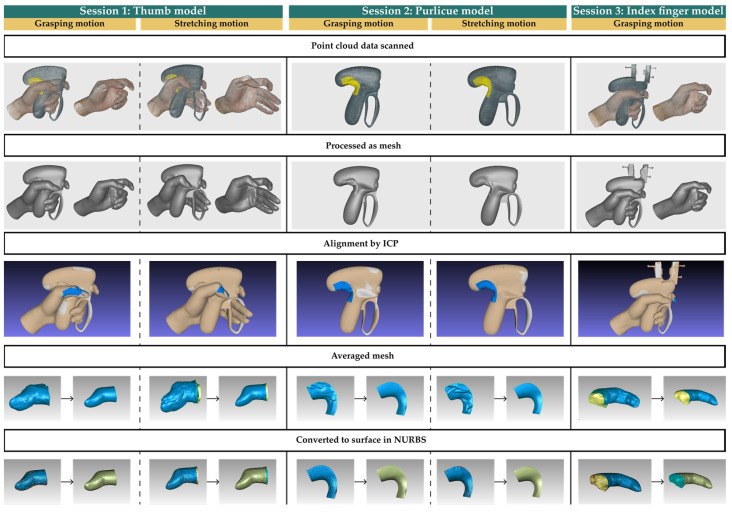
Design procedure of the IH.

Documented analysis relating to the tool operation was conducted for the laparoscopic dissectors. Figure 2 schematically shows the way in which the dissector is manipulated. Power actions are used for opening and closing the end-effectors of the forceps in order to complete the dissection or spreading task. Index fingers are usually used for rotating or pressing the knob in order to emit energy such as sintering and coagulation. In order to obtain the maximal range of hand movements, a 3D scanner (Artec Space Spider, Artec 3D, Senningerberg, Niederanven, Luxembourg) was adopted to capture the surface geometry of the maximal hand positions. A group of 21 volunteers (16 males and 5 females) participated in this scanning experiment, whose suitable size of medical examination gloves was medium (7 and 7.5) [47]. Their anthropometric characteristics were collected and they are reported in Table 1. The scanning procedure involved three sessions, which are described in the following sections (Figure 3).

- Session 1. In order to create additional support for the thumb, two positions for the maximal hand movement with the OH, power grasp, and stretch were required to be held by the volunteers and were scanned. Since light cannot enter through the small gaps, comprehensive models of the thumb could not be captured by the optical scanner. Therefore, another posture maintaining the position of the thumb without the OH was also scanned. The two types of point cloud data scanned were aligned and processed to recover the shape and position of the thumb.
- Session 2. In order to avoid the collision between the thumb metacarpophalangeal articulation and the handle surface during operation, more space was needed around the purlicue. Clay modeling was used to create the physical interface between the handle and the purlicue. The molded clay around the blanks exposed was intended to create interaction surfaces of the prototype, and each volunteer created two clay sculptures with the grip posture in the power grasp and stretch positions. The clay sculptures were scanned to convert physical models to point cloud data, and the interfaces between the purlicue and the handle were obtained.
- Session 3. A new prototype based on the OH was designed to establish the position of the activation button for coagulation, which was manipulated by the index finger when the handle was power grasped. The prototype included a vernier caliper connected to the functional button, which was able to move with the position of the index finger. Each volunteer was required to hold the prototype and place the index finger in a comfortable position pressing the button. Two types of models were captured, the gripping posture with the prototype and the shape of the index finger without the prototype. The two scans were aligned and processed to recover the shape and position of the index finger. In addition, the reading of the vernier caliper indicated the distance the button needed to move, which was recorded to identify the appropriate position of the activation button.

### 2.2. Description of the Evaluated Tool Design

The point cloud data of the three sessions captured in the scanning of Section 2.1.2 were processed as mesh after global registration, outlier removal, sharp fusion, small-object filtering, hole filling, and mesh simplification in Artec Studio 17 Professional (Artec 3D, Senningerberg, Niederanven, Luxembourg). The corresponding meshes in each group were aligned using iterative closest point (ICP) alignment in MeshLab (Visual Computing Lab of ISTI-CNR, Pisa, Pisa, Italy). Three meshes were averaged using Geomagic Wrap (Artec 3D, Senningerberg, Niederanven, Luxembourg) and were converted to smooth surfaces in NURBS, which can be applied to product design. The modeling process of IH was conducted in Rhinoceros 7 (Robert McNeel and Associates, Seattle, WA, USA) by creating blend surfaces with G3 continuity between the surface of the OH and the interface of the index finger, as well as the thumb. The design process is illustrated in Figure 3.

**Figure 4:**
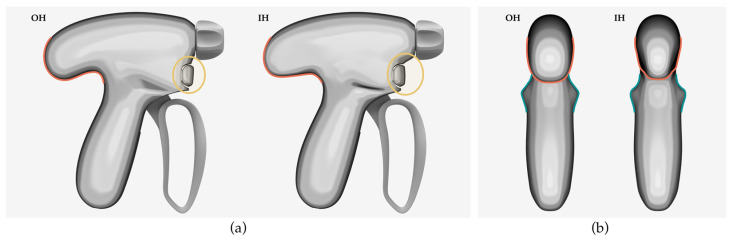
(**a**) The lateral view of the geometric morphometric comparison between the OH and IH; (**b**) The posterior view of the geometric morphometric comparison between the OH and IH.

The geometric morphometric comparison between the OH and the IH is shown in Figure 4. The first change made to the IH was to create a better fit support area to provide support for the thumb when exerting force, which can reduce high-pressure zones of the thumb and prevent unnecessary loss of flexion force. The second change involved camber adjustment to better fit the purlicue, and an inward shape at the tail of the handle to provide an avoidance area to reduce friction and collision between the thumb metacarpophalangeal articulation and the handle. The third improvement was to add the back-moved location and the downward-sloping shape of the activation button to make it more suitable for the natural grasp posture of the index finger and to improve the operational flexibility.

**Figure 5:**
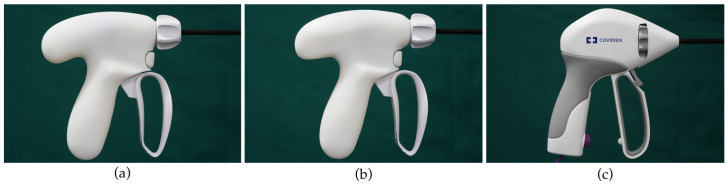
(**a**) The OH used in the evaluation; (**b**) The IH used in the evaluation; (**c**) The CH used in the evaluation.

Only the handles of the laparoscopic dissectors were prototyped by 3D printing with an ABS-like photopolymer (Covestro’s Somos^®^ ProtoGen 18420). Thus, the ergonomic assessment in this research was exclusively attributed to the ergonomic design of the handle. In order to observe the ergonomic performance compared to commercial instruments on the market, a widely used laparoscopic tool, Ligasure Maryland forceps (Medtronic Covidien Minneapolis, MN, USA), labeled as CH, was also used in the evaluation (Figure 5).

### 2.3. Volunteers Taking Part in the Assessment

The study population was divided into two groups. The first group comprised 16 novices (8 male and 8 female) who were selected from the students of Hunan University. The second group was composed of 14 surgeons (7 male and 7 female). Participants with musculoskeletal disorders were not included. The selected participants were informed of the objective and practical aspects of the experiment. After informed consent was signed by each participant, they completed an initial questionnaire to obtain their demographic and anthropometric information, which is presented in Table 2.

### 2.4. Task Description

**Figure 6:**
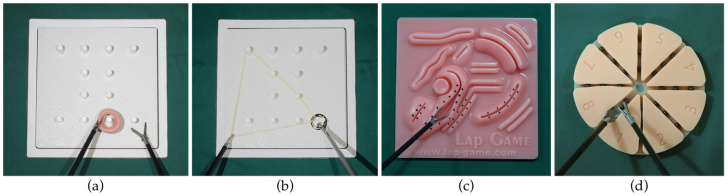
(**a**) Task 1 (T1) conducted in the evaluation; (**b**) Task 2 (T2) conducted in the evaluation; (**c**) Task 3 (T3) conducted in the evaluation; (**d**) Task 4 (T4) conducted in the evaluation.

Training boxes were used to develop tasks simulating the real conditions of laparoscopic operations instead of actual surgical interventions to obtain the necessary conditions for repeatability and reproducibility [23]. In this study, four tasks were included for the ergonomic assessment (Figure 6) based on the work presented by the authors of [24,26].

T1 consisted of twelve movements of a ring-type object on a pegboard. Each grasp was required to last 10 s. When grasping, participants were asked to simultaneously press the activation button with their index fingers. In T2, one side of an elastic band was placed on the pegboard and fixed on the side of the participant’s dominant hand, and participants were asked to place specific pegs on the other side sequentially. T3 consisted of twenty points on a laparoscopic simulator 3D-stitching module, which needed to be inserted by the tip of the dissectors. Participants were required to open the end-effector with a pushing force and kept exerting the force for 5 s for each point sequentially. T4 included the transformation of twenty-four chickpeas on a multi-wound suture model. Participants were asked to open the wound using their non-dominant hand and transfer the chickpeas to a small box using their dominant hand.

**Figure 7:**
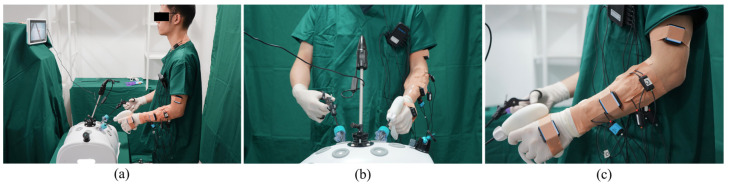
(**a**) A surgeon performing T3 using an IH in his left hand; (**b**) The front view of the surgeon’s operation; (**c**) The location of EMGs and IMUs on the surgeon’s left arm.

In all tasks, the laparoscope remained fixed, so the volunteers were only concerned with the use of the instruments requiring the non-dominant hand. The volunteers were located on a height-adjustable platform in front of the training box to modify the height of the subject (Figure 7). Each volunteer had to complete one test, comprised of four tasks, with all handles, the OH, IH, and CH. The order of the instruments used during the test was randomly changed to avoid the learning effect.

### 2.5. Objective Survey

Evaluation with respect to muscle effort, goniometry analysis, efficiency, and effectiveness was carried out by means of an objective survey. The muscle effort was measured using sEMG (PhysioLab, Ergoneers, Germany). Six electrodes were placed on the participants’ left arm on the following muscles: extensor carpi ulnaris (EMG1), flexor pollicis brevis muscle (EMG2), flexor carpi radialis (EMG3), brachioradialisn (EMG4), extensor digitorum communis (EMG5), and trapezius (EMG6). The raw signal was recorded by EMG electrodes with a sampling frequency of 2 kHz. The maximum voluntary contraction (MVC) was obtained from each participant at the beginning of the experiment according to [48]. The muscle load was expressed as the ratio of the root mean square (RMS) amplitude recorded during the task completion to that obtained during MVC. The rotation of the upper extremities was measured by inertial measurement units (IMUs) (BWT61CL IMU Sensor, Witmotion, Shenzhen, China). The goniometric analysis during the test included the measurement of the following angles: wrist extension (G1), wrist flexion (G2), wrist ulnar deviation (G3), wrist radial deviation (G4), forearm protonation (G5), forearm supination (G6), shoulder abduction (G7), and shoulder adduction (G8). Additionally, the angles were compared with the limits provided by the REBA [49], ISO 11226 [45], and ISO 11228 standards [50]. The total execution time (TT) for each task was measured as the efficiency of each handle. The summation of the number of achievements for completing each task was considered the effectiveness of each handle.

### 2.6. Subjective Survey

Questions related to the difficulty of using each handle in each task, the preference of using the handle to repeat the tasks again (Q1), and the most painful handle to use (Q2) were asked to collect information regarding the participants’ attitudes. Questions including those related to pain, intuitiveness, comfort, precision, stability, and overall satisfaction according to [9,51] were used to profile the subjective handle evaluation. All of the ratings were evaluated using a visual analogue scale (VAS) that consisted of a line 10 mm in length with appropriate written anchors at the extremes (e.g., “not painful at all” to “Extremely painful”). Thereafter, the participants could freely express everything they thought about the three handles.

### 2.7. Statistical Analysis

Summary statistics for EMG, motion analysis, subjective ratings, TT, and achievement numbers were analyzed using SPSS software version 26.0 (IBM SPSS Inc., Chicago, IL, USA). The Shapiro–Wilk test was applied to check whether the data met the required assumptions of a parametric test. Data from TT, EMG, and motion analysis were analyzed with parametric tests and described by the mean and standard deviation (SD). One-way ANOVA was applied to obtain the differences between the handles. Subjective ratings were analyzed with non-parametric tests. The Kruskal–Wallis test on ranks was applied to compare subjects’ feedback relating to the handles. Significance values were adjusted using the Bonferroni correction for multiple tests, and the variables were described by the median and interquartile range (IQR). Data relating to the number of achievements were processed using Wilson’s method to obtain the confidence intervals of the percentages. Participants’ preference was processed using Cochran’s Q test. Moreover, the comparisons between novices and surgeons were evaluated using Student’s t-test when there was a normal distribution, or the Mann–Whitney U test when the data did not fulfill the condition. For all tests, *p* < 0.05 was considered to be statistically significant.

## 3. Results

### 3.1. Objective Survey

**Figure 8:**
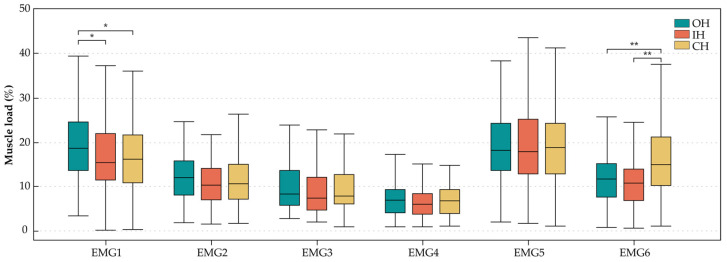
Box plot diagrams of EMG results of the handles. * *p* < 0.05, ** *p* < 0.01.

Figure 8 shows the results of the muscle load of EMG1, EMG2, EMG3, EMG4, EMG5, and EMG6 under various tasks. When using the OH and IH, muscle load was higher in EMG 1 and EMG5. When using the CH, EMG1, EMG5 and EMG6 were classified into the group with the greatest muscle load. No significant difference was found among the three handles for all muscles except EMG1 and EMG6. The OH resulted in a significantly higher muscle load than the IH and CH in EMG1. The CH caused a significantly higher muscle load compared with the OH and IH in EMG6.

The goniometric analysis results were within the limits established, and the differences among the handles were significantly different (Table 3). G1 and G7 of the OH had a tendency to require a rather small wrist extension (IH: *p <* 0.001, CH: *p* < 0.001) and shoulder abduction (IH: *p* = 0.021, CH: *p* < 0.001). According to the post hoc test results, G2, G3, G4, G5, G6, and G8 of the IH were the smallest compared to the OH (*p* = 0.395, *p* < 0.001, *p* = 0.050, *p* = 0.919, *p* = 0.081, *p* < 0.001) and the CH (*p* < 0.001, *p* = 0.046, *p* < 0.001, *p* < 0.001, *p* < 0.001, *p* < 0.001).

**Figure 9:**
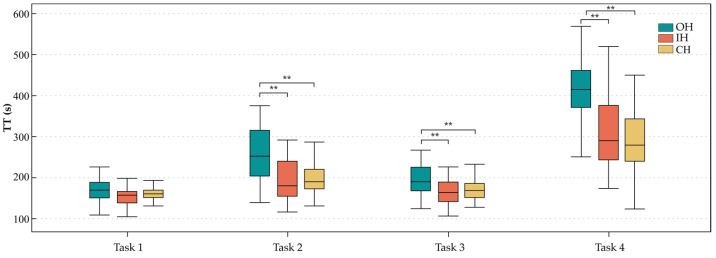
Box plot diagrams of total execution time of the tasks. ** *p* < 0.01.

Statistically, the effect of the handles on TT was significant in all tasks except T1 (Figure 9). Based on the post hoc results, the IH and CH were not significantly different in T2, T3, and T4, which were significantly lower compared with the OH.

**Figure 10:**
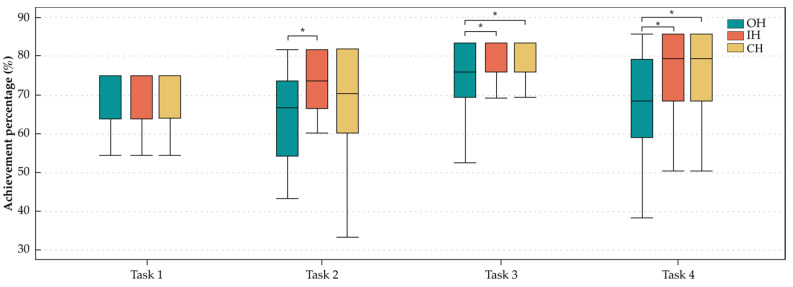
Box plot diagrams of the achievement percentage results from the tasks. * *p* < 0.05.

The achievement percentage was significantly affected by the handles in T2, T3, and T4, and the results obtained are shown in Figure 10. In T2, the IH showed a significantly higher achievement percentage compared only with the OH. In T3 and T4, the values of the IH and CH were similar and both were higher than that of the OH.

### 3.2. Subjective Survey

**Figure 11:**
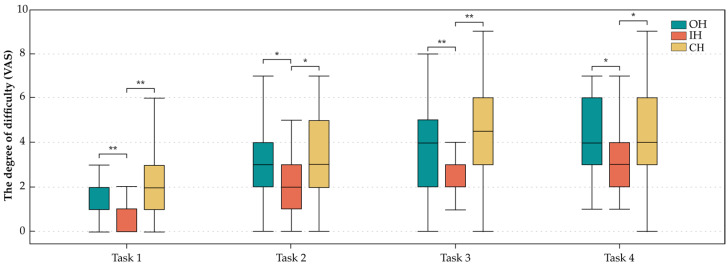
Box plot diagrams of results of the degree of difficulty of each task. * *p* < 0.05, ** *p* < 0.01.

The statistical results of the answers to the questionnaire provided an opinion on the degree of difficulty in completing the tasks, which is shown in Figure 11. The values of the IH were significantly lower than those of the OH and CH for all tasks, which showed similar results. The reported degree of difficulty in T1 did not differ between novices and surgeons. However, the values were significantly different in T2 when working with the OH (*p* = 0.032) and CH (*p* = 0.001), and surgeons reported lower ratings than novices. Surgeons reported a significantly lower degree of difficulty with all three handles in T3 (OH: *p* = 0.012, IH: *p* = 0.012, CH: *p* = 0.007) and T4 (OH: *p* = 0.006, IH: *p* = 0.001, CH: *p* = 0.001).

**Figure 12:**
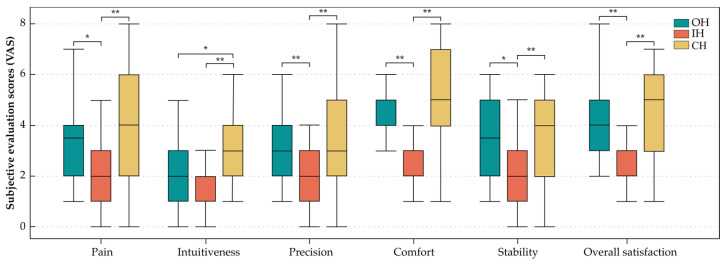
Box plot diagrams of results of the subjective evaluation after completing the tasks. * *p* < 0.05, ** *p* < 0.01.

The effect of the handle type on the subjective handle evaluation values related to pain, intuitiveness, comfort, precision, and overall satisfaction was significant, as shown in Figure 12. The OH and IH had similar reported intuitiveness results, which were significantly lower than that reported for the CH. For the values of pain, precision, comfort, stability, and overall satisfaction, the IH showed markedly lower ratings compared to the other two handles. Based on the pairwise comparison between novices and surgeons, significantly higher ratings for precision, stability, and overall satisfaction were reported by novices but only when working with the CH (*p* = 0.008, *p* = 0.042, *p* = 0.010).

In the answer to Q1, all participants expressed that the IH was the handle that they would choose if they had to repeat the same tasks for several hours. The answers to Q2 revealed that no participants considered the IH as the most painful tool, and the results for the OH (40%) and the CH (60%) were similar (*p =* 0.220). In addition, 64.3% of surgeons opted for the OH and 35.7% opted for the CH, and the difference was not significant (*p =* 0.571). However, 81.3% of the novices selected the CH as the most painful instrument, which was significantly greater than for the OH (18.8%, *p =* 0.007).

## 4. Discussion

The main objective of this work was to develop an ergonomic handle for a laparoscopic dissector with the considerations of anthropometry and dynamic hand positioning to reduce the risk of MSD during LS. In this study, the new handle, the IH, was designed using 3D anthropometric data obtained from scanning the maximal positions of hand movement. Compared with the commercial instrument, the main difference was that the IH achieved additional support for the thumb, a better fit to the purlicue, and a more flexible grasp for the index finger. This avoids force loss when the thumb is exerted, reduces small high-pressure zones for the purlicue, and makes the pressing of the activation button more effective. The new design was also validated by demonstrating its superiority over a commercial dissector and an original handle before improvement, with respect to biomechanical and psychophysical aspects. The assessment trials demonstrated the differences among the three handles, and they are discussed in the following paragraphs.

The significance of muscle load among the handles was observed only in the extensor carpi ulnaris and trapezius, similar to the findings in [5,6,52]. Regarding the extensor carpi ulnaris, the OH presented the largest muscle load, and the CH caused the greatest muscle load in the trapezius. The muscle load corresponded with the result of the goniometric study, where the shoulder adduction and abduction were the largest when using the CH, which may be the reason for the large muscle load of the trapezius.

Generally, the most fatigued muscles were the extensor carpi ulnaris and the extensor digitorum communis whilst using the handles. The fatigue of the extensor carpi ulnaris was mainly caused by the wrist deviation, and the fatigue of the extensor digitorum communis was mainly caused by the exertion of the fingers in order to open and close the end-effectors, which are the motions most often undertaken during dissection tasks. This finding is similar to the result in [22]. In addition, slight but not significant superiority in terms of less fatigue of the flexor pollicis brevis muscle was obtained for the IH, although the support around the thumb area was designed to reduce the force loss during exertion. This may be because the grasping and stretching motions in the tasks conducted in this research were performed alternately, and the duration of each posture hold was not long enough to cause a significant difference.

Although there were differences in the goniometric study of the angles of the wrist, forearm, and shoulder, the values of the angles were within the acceptable range of motion for all handles. The results also indicate that the grip design of the IH and OH can contribute to a natural posture compared with that of the CH during the dissection tasks.

Generally, the performance of the IH in the TT was better than that with the other two handles. The TT of the IH was slightly shorter compared to that of the CH in all tasks except T4. The reason for the faster TT of the CH can be explained by the end-effectors of the OH and IH being different from that the CH, and they are removed from other commercial dissectors.

The parameters measured for effectiveness showed the IH had the highest achievement percentage among the three handles, although the difference was not significant in T1, which may be because the task was not difficult for any of the handles tested.

One of the most significant results was that the degree of difficulty reported with the IH was significantly lower compared with that of the other two handles, regardless of the fact that the results of the tasks indicate that the use of the IH made the tasks simple. The values of the OH, which were slightly but insignificantly better than those of the CH, may have been due to the assistance provided by the support area designed for thumb, although it did not fit well. The intuitiveness values of the IH and OH both showed better results than that of the CH, which indicated that the redesign of the geometry of the handle made it easier to use for novices. Generally, the IH presented the best subjective evaluation results in the simulated surgical tasks tested, which corresponded with the results of Q1; all subjects preferred the IH if they had to repeat the tasks. Reasons were collected and revealed that the size of the IH was smaller and rounded, which made it more comfortable and flexible to hold. It was noted that most participants addressed the advantages of the support area designed for the thumb, which made it easier to exert force when carrying out the stretching task. In terms of the most painful tool, the CH had slightly but insignificantly more votes than the OH. The reason for choosing the CH was mainly due to the lack of a support point for the thumb to exert force, which resulted in poor usability. In addition, subjects who chose the OH reflected that the OH was a little wide to hold, and there were small high-pressure zones in the hand when operating the handle. That may be because the OH was based on the static gripping hand position, the movement of the thumb metacarpophalangeal articulation when finger exertion took place was not squared up. Thus, high-pressure zones occurred due to the small avoidance space of the thumb and purlicue area; there was also a poor fit between the hand and the handle surface.

Since the previous experience of surgeons in using these instruments can provide important insights for discussion and future work, comparisons in terms of subjective values were conducted between novices and surgeons. The degree of difficulty reported by novices was significantly higher than that reported by surgeons in several specific trials. This may because surgeons become familiar with the simulation task more quickly and are more familiar with the use of laparoscopic tools. The values of subjective evaluation of the OH and IH showed no significant difference between the two groups. However, the surgeons reported better feedback of precision, stability, and overall satisfaction with the CH, whose type was the most often used in their previous surgery. Moreover, there was no significant difference in the most painful tools between the CH and OH for surgeons, but more novices thought the CH was the worst handle. This may be affected by the negative and positive attitude to the CH in surgeons’ previous experience, and this effect was also reported by the authors of [23]. It is worth mentioning that the new handle, the IH, was the preferred laparoscopic tool among the three handles for the surgeons.

The new design of the handle was positively evaluated by the participants of this study with regard to the use of a series of simulating surgical tasks. Both subjective and objective results showed the significant superiority in the feasibility and usability of the new handle compared to the handle before improvement and the commercial laparoscopic tool. To the best of our knowledge, this methodology for the creation of a new handle is the first to apply 3D hand anthropometric data to a surgical instrument, involving dynamic hand positions and comprehensive anthropometric measurements. The application of this design methodology was highly recognized by not only novices but also surgeons.

However, the results of this study are limited for several reasons. Primarily, the data of only 21 volunteers were included in this study, while in order to obtain more representative results, a large volume of 3D hand anthropometric data is necessary. In the future, a 3D hand anthropometric database is required to be built, and the effect of hand size and gender can be explored in depth. Furthermore, in order to maintain the repeatability and safety of the experiment, the tasks for the ergonomic assessment were performed in a simulated environment instead of in an actual operating theater. This introduces uncertainty regarding how the behavior of the subjects would be affected under real conditions. Thus, real surgical interventions and a longer duration of use of the instruments are necessary to understand the behavior of the handles. Another limitation of this assessment was the lack of pressure distribution in the hand with the contact of the handle. Pressure data can provide an objective reflection on high-pressure zones that are related to pain in the hand. Therefore, pressure analysis will be included in future research.

## 5. Conclusions

This study piloted a methodology of a laparoscopic dissector handle design using 3D hand anthropometric data of several dynamic hand positions. A new handle was designed by applying this reverse engineering methodology to implement improvements that provided additional support for the thumb, a better fit to the purlicue, and a more flexible grasp for the index finger. In terms of the ergonomic assessment, significant superiorities were observed in the new handle with respect to both objective survey and subjective evaluation. The positive results verify the significant implications for the handle design for a laparoscopic dissector and contribute to the methodology of hand tool design based on 3D anthropometric data and dynamic hand positions. The hand data used in this research are expected to be expanded in order to facilitate the universal and customized design of laparoscopic handles as well as other types of hand tools.

## Figures and Tables

**Table 1:** Anthropometric characteristics of the participants of the scanning experiment.

Variable (n = 21)	Mean	SD	Minimum	Maximum
Age (years)	23.0	1.9	19.0	27.0
Height (cm)	173.9	5.7	167.0	185.0
Weight (kg)	67.9	10.3	55.0	87.0
Hand length (mm)	190.7	7.2	180.6	204.6
Hand circumference (mm)	194.1	0.9	186.5	206.0
Glove size	7.4	0.2	7.0	7.5

Data are shown as the mean and standard deviation. cm = centimeters; mm = millimeters; kg = kilograms; SD = standard deviation.

**Table 2:** Distribution and anthropometric characteristics of participants.

Variables	Surgeons		Novices	
	Mean	SD	Mean	SD
Age (years)	28.142	3.900	25.000	3.864
Height (cm)	169.429	8.355	169.434	8.594
Weight (kg)	64.750	11.869	62.563	13.256
Hand length (mm)	186.817	11.514	186.358	11.015
Hand breadth (mm)	79.323	5.075	79.651	6.735
Palm length (mm)	109.3281	7.774	106.271	7.814
Hand thickness (mm)	27.621	3.115	27.429	2.499
Hand circumference (mm)	188.214	12.330	175.806	43.035
Wrist circumference (mm)	155.643	11.230	135.173	48.735
Medical examination glove size	7.180	0.460	7.250	0.500

Data are shown as the mean and standard deviation. cm = centimeters; mm = millimeters; kg = kilograms; SD = standard deviation.

**Table 3:** Statistical results from the goniometric angles.

Variables	OH		IH		CH		F	*p*	Limit		
	Mean	SD	Mean	SD	Mean	SD			REBA	ISO 11226	ISO 11228
G1	12.134	8.125	12.823	8.523	12.874	8.138	75.988	0.000 **	15	90	40
G2	11.703	8.454	11.799	8.189	12.596	8.359	89.004	0.000 **	15	90	45
G3	9.397	6.206	9.033	5.939	9.380	5.898	40.831	0.000 **	n/a	30	n/a
G4	8.832	5.915	8.689	5.808	9.204	6.157	33.444	0.000 **	n/a	20	n/a
G5	9.511	6.712	9.529	6.663	10.036	6.913	79.050	0.000 **	n/a	90	n/a
G6	4.180	3.234	4.087	3.443	4.543	3.534	58.582	0.000 **	n/a	60	n/a
G7	6.236	4.879	6.351	4.758	6.407	4.944	8.005	0.000 **	n/a	60	n/a
G8	7.095	5.578	6.479	5.539	8.026	5.736	555.962	0.000 **	n/a	0	n/a

Data are shown as the mean and standard deviation. The F and *p* values of the ANOVA are shown. SD = standard deviation; n/a = not available. ** *p* < 0.01.

## Data Availability

The data presented in the present study are available on request from the corresponding author.
